# Morphologic prognostic factor for thoracoabdominal aortic dilation after acute type A dissection repair

**DOI:** 10.1093/icvts/ivae063

**Published:** 2024-04-08

**Authors:** Yuichiro Kitada, Homare Okamura, Hidenari Hasui, Kei Akiyoshi, Yohei Nomura, Hideo Adachi

**Affiliations:** Department of Cardiovascular Surgery, Nerima Hikarigaoka Hospital, Tokyo, Japan; Department of Cardiovascular Surgery, Nerima Hikarigaoka Hospital, Tokyo, Japan; Department of Cardiovascular Surgery, Nerima Hikarigaoka Hospital, Tokyo, Japan; Department of Cardiovascular Surgery, Nerima Hikarigaoka Hospital, Tokyo, Japan; Department of Cardiovascular Surgery, Nerima Hikarigaoka Hospital, Tokyo, Japan; Department of Cardiovascular Surgery, Nerima Hikarigaoka Hospital, Tokyo, Japan

**Keywords:** Aortic dissection, Thoracoabdominal aortic dilation, Aortic morphology

## Abstract

**OBJECTIVES:**

Risk factors for late-term aortic dilation after acute type A aortic dissection repair have not been well examined. The goal of this study was to determine the relationship between the abdominal aortic true lumen location and thoraco-abdominal aortic dilation after surgical repair for acute type A aortic dissection.

**METHODS:**

Patients who were preoperatively diagnosed with acute type A aortic dissection between April 2014 and July 2022 were included in this study. We evaluated the renal artery-level dissected aortic morphology and classified the study population into 2 groups: the ventral (those with the true lumen located on the ventral side) and the dorsal (other patients not assigned to the ventral group) groups, based on the location of the true lumen. Aortic dilation was defined as thoraco-abdominal aortic expansion ≥5 mm on 1-year postoperative computed tomography images.

**RESULTS:**

We examined 49 surgical patients who were assigned to the ventral (n = 22) and dorsal (n = 27) groups. The number of patients with ≥5 mm thoraco-abdominal aortic dilation after the operation was significantly higher in the ventral group than in the dorsal group (90.9% vs 51.9%, *P *=* *0.009). The multivariable logistic regression analysis showed that the ventral type was an independent prognostic factor for thoraco-abdominal aortic dilation after the operation (odds ratio, 6.01; 95% confidence interval, 1.56–23.77; *P *=* *0.009).

**CONCLUSIONS:**

The location of the true lumen of the abdominal aorta in acute type A aortic dissection may be a prognostic factor for thoraco-abdominal aortic dilation after surgical repair.

## Graphical abstract

**Figure:**
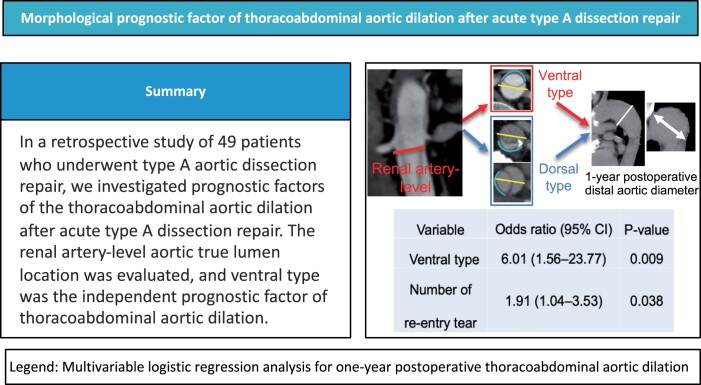

## INTRODUCTION

Over the past few decades, surgical outcomes of acute type A aortic dissection (ATAAD) have improved [1]. However, in some patients with a residual patent false lumen (FL), reoperation for distal aortic aneurysmal dilation is required. Several studies have reported that a patent FL is an independent predictor of secondary aortic enlargement [2–4]. Kimura *et al.* reported postoperative FL patency in 62% of patients following an emergency surgery, and 12.1% of patients with a patent FL had undergone distal reoperations, 6.1% of whom died of aortic rupture [5]. Identifying the risk factors for thoraco-abdominal aortic dilation could lead to the development of therapeutic agents and improve late outcomes of a first aortic repair for ATAAD.

Although guidelines recommend postoperative imaging surveillance, adherence to the recommended guidelines is poor and associated with mortality and reintervention following ATAAD repair [6–8]. Identification of patients with risk factors for late-term aortic expansion or rupture, via strict surveillance, may improve late outcomes after ATAAD repair.

Recently, various risk factors have been identified for the prediction of descending aortic enlargement after uncomplicated type B aortic dissection (TBAD) [9–19]. However, late-term thoraco-abdominal aortic dilation after aortic repair for ATAAD has not been thoroughly evaluated. Salier *et al.* suggested that the number of intercostal arteries branching from the FL is an independent predictor of late adverse events in acute uncomplicated TBAD [16]. In addition, the researchers considered that the small outflow from the FL increased FL pressure and contributed to aortic dilation. Thus, thoraco-abdominal aortic enlargement may be affected by the location of the true lumen (TL), which affects the number, size and location of re-entry tears formed by visceral, intercostal or lumber branches. Based on these findings, we considered that the TL location, that is, the lumbar branches mainly from the TL or FL, may influence poor late-term thoraco-abdominal aortic dilation.

Our goal was to evaluate the abdominal aortic TL location as a simple and precise prognostic factor for the identification of patients who are at high risk for late thoraco-abdominal aortic dilation after proximal aortic repair for ATAAD.

## MATERIALS AND METHODS

### Ethics statement

The study protocol was approved by the institutional review board of Nerima Hikarigaoka Hospital on 30 May 2023 (No. 22090802), and the need for individual patient consent was waived due to the retrospective nature of the study.

### Study population

We reviewed our institutional databases. A total of 187 patients who underwent aortic surgery for ATAAD between April 2014 and July 2022, were identified. In our hospital, we performed preoperative and 1-week postoperative computed tomography (CT) scans with contrast enhancement, and depending on the patient’s condition, a 1-year postoperative CT scan with or without contrast enhancement. The patient inclusion criteria were as follows: available preoperative, 1-week postoperative and 1-year postoperative CT findings; patent FL before surgery; and an abdominal aortic dissection. A total of 138 patients were excluded because they died before the 1-year postoperative CT scan; FL thrombosis occurred before the operation; no contrast-enhanced CT was performed before surgery; they were lost to follow-up before the 1-year postoperative CT; they received additional treatment within 3 months after the first surgery for distal aortic rapid enlargement or stent-induced new entry caused by a frozen elephant trunk (FET); the primary entry occurred at the descending aorta or at an unknown location; and no dissection occurred in the abdominal aorta. Overall, 49 patients were included in this study, and their CT images and medical records were reviewed.

### Study design

**Figure 1:**
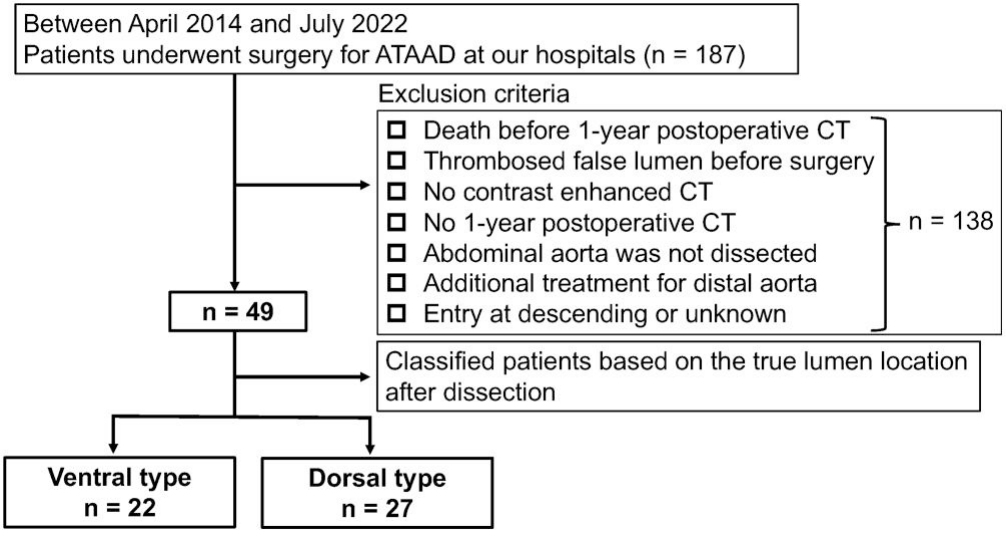
Patient flow diagram. ATAAD: acute type A aortic dissection; CT: computed tomography.

Occasionally, aortic dissection proceeds helically from the proximal descending aorta to the aortic bifurcation; thus, 1 assessment point should be identified to determine the TL location. Therefore, owing to the ubiquity and understandability of the TL location in preoperative contrast-enhanced CT scans, we evaluated the TL location at the level of the distal renal artery and classified the patients into 2 groups based on the TL location (Fig. 1).

To evaluate thoraco-abdominal aortic dilation, we measured each segment of the preoperative and the 1-year postoperative thoraco-abdominal aortic diameter at zone 3, from the fourth thoracic to the first lumber level and identified the maximum dilated segment. We defined the cutoff value of aortic dilation as ≥5 mm expansion of the aortic diameter 1 year postoperatively compared to the preoperative aortic diameter at the same level. A 1-year follow-up CT scan was defined as the plane or contrast-enhanced CT scan performed between 9 and 15 months postoperatively.

We evaluated the relationship between the abdominal location of the TL and the 1-year postoperative thoraco-abdominal aortic dilation.

### Image analysis

**Figure 2:**
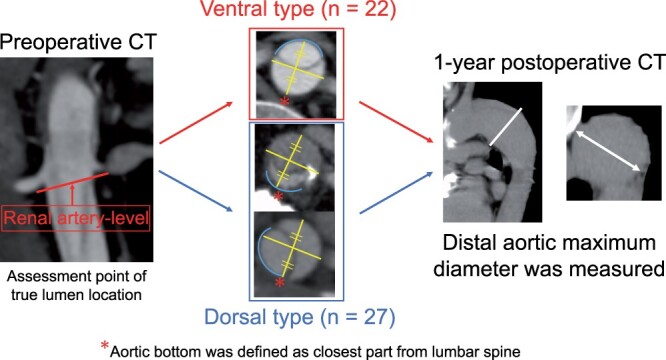
True lumen location evaluation of the dissected abdominal aorta and 1-year postoperative aortic expansion. CT: computed tomography.

The Digital Imaging and Communications in Medicine standard data for eligible patients were extracted from the database for further analysis. One researcher performed the image analyses using Ziostation 2 (Ziosoft, Inc, Tokyo, Japan). The aortic dimensions were measured from multiplanar reformatting using appropriate perpendicular planes, and we measured the aortic dimension at each segment of the thoraco-abdominal aorta. The ventral type was defined as the TL extending completely in the ventral side of the aorta. This designation indicated that the nondissected part of the TL wall was located completely in the upper half of the semicircle. In contrast, the dorsal type was defined as the TL in the dorsal side or not completely in the ventral side. The bottom of the aorta was defined as the region closest to the lumbar spine (Fig. 2). A completely thrombosed thoraco-abdominal aortic FL was defined as a condition in which the FL from the distal anastomosis or FET edge to the terminal aorta was completely thrombosed. In contrast, a patent FL was defined as no thrombosis identified throughout the FL. A spiral dissection pattern was defined as a change in the position of the middle of the FL (compared with the TL) by a minimum of 90 degrees, at any level between the left subclavian artery and the aortic bifurcation. The primary entry was identified using preoperative CT scans and classified into 2 types based on the location and on the inner and outer types of curvatures. The location of the re-entry tears and the number of re-entry tears were identified from postoperative contrast-enhanced CT scans. The TL was configured as concave or convex, and we defined patients with concave TLs as those who had concave-shaped TLs at any level between the left subclavian artery and the aortic bifurcation.

### Surgical procedure and postoperative management

An ascending aortic or aortic arch replacement with or without FET was performed in all patients within 24 h of onset of ATAAD. Arch replacement was defined as replacement of any supra-aortic vessel during the operation. Primary entry was excluded in all patients. A commercially available FET graft (J Graft FROZENIX; Japan Lifeline CO, Ltd, Tokyo, Japan) was used in patients with an FET.

### Statistical analyses

Categorical variables are expressed as frequencies and percentages, and continuous variables are expressed as mean ± standard deviation for normally distributed variables and as median (interquartile range) for non-normally distributed variables. The Fisher exact test was used to compare categorical variables. Normally distributed continuous variables were compared using the Student *t*-test, and the non-parametric Mann–Whitney U test was used when at least 1 group had non-normally distributed variables. The Shapiro–Wilk test was performed to evaluate whether continuous variables were normally or non-normally distributed. A logistic regression analysis was performed to analyse the prognostic factor of 1-year postoperative thoraco-abdominal aortic dilation. First, univariable logistic regression analysis was conducted to separately calculate odds ratios (ORs) for each covariate with 1-year postoperative thoraco-abdominal aortic dilation. Variables with a *P*-value < 0.30 in an univariable analysis were subjected to a multivariable logistic regression analysis. A backward stepwise multivariable logistic regression analysis was performed using variables identified in the univariable analysis. The cut-off value for classification was 0.05 for the backward stepwise selection. Due to the small study population, adjustments were not made. The total sample size of 49 patients provided a post hoc power of 96.0% for the detection of differences in the logistic regression analysis for the 1-year postoperative thoraco-abdominal aortic dilation; the assumed treatment effect was 10.95.

All statistical analyses were performed using SPSS software version 29 (IBM Corp., Armonk, NY, USA). A *P*-value < 0.05 was considered statistically significant.

## RESULTS

### Baseline patient characteristics and operative data

Of the 49 patients, 22 (44.9%) and 27 (55.1%) were in the ventral and dorsal groups, respectively. The preoperative baseline characteristics of the study population are shown in Table 1. There were no significant differences in the patient characteristics or primary entry sites between the groups. The intraoperative data are shown in Table 2. Root surgery was performed in 7 (14.3%) patients and was significantly higher in the ventral group than in the dorsal group as derived from Fisher’s exact test (27.3% vs 3.7%, *P *=* *0.036). An FET was used in 33 (67.3%) patients; however, no significant difference in FET usage and in its size or length was observed.

**Table 1:** Study population baseline characteristics

Variable	Total	Ventral	Dorsal	*P*-value
	(*n *=* *49)	(*n *=* *22)	(*n *=* *27)	
Age, years	55.6 ± 9.5	55.7 ± 10.5	55.6 ± 8.9	0.97
Males, *n* (%)	33 (67.3)	15 (68.2)	18 (66.7)	1.000
Body surface area, m^2^	1.77 ± 0.23	1.74 ± 0.21	1.75 ± 0.25	0.88
Hypertension, *n* (%)	30 (61.2)	14 (63.6)	16 (59.3)	0.77
Diabetes, *n* (%)	0 (0)	0 (0)	0 (0)	1.000
Hyperlipidaemia, *n* (%)	3 (6.1)	2 (9.1)	1 (3.7)	0.58
COPD, *n* (%)	0 (0)	0 (0)	0 (0)	1.000
Chronic kidney disease^a^, *n* (%)	4 (8.2)	2 (9.1)	2 (7.4)	1.000
Marfan syndrome, *n* (%)	1 (2.0)	1 (4.6)	0 (0)	0.45
Primary entry location				
Ascending aorta, *n* (%)	23 (46.9)	12 (54.6)	11 (40.7)	0.40
Aortic arch, *n* (%)	25 (51.0)	10 (45.5)	15 (55.6)	0.57
Outer curvature, *n* (%)	37 (75.5)	15 (68.2)	22 (81.5)	0.33
Inner curvature, *n* (%)	12 (24.5)	7 (31.8)	5 (18.5)	0.33
Visceral branches arising from the false lumen				
Celiac artery, *n* (%)	24 (49.0)	7 (31.8)	17 (63.0)	0.045
Superior mesenteric artery, *n* (%)	20 (40.8)	4 (18.2)	16 (59.3)	0.008
Left renal artery, *n* (%)	39 (79.6)	16 (72.7)	23 (85.2)	0.31
Right renal artery, *n* (%)	12 (24.5)	4 (18.2)	8 (29.6)	0.51
Inferior mesenteric artery, *n* (%)	23 (46.9)	5 (22.7)	18 (66.7)	0.004
Number of visceral branches arising from the false lumen				
0, *n* (%)	3 (6.1)	2 (9.1)	1 (3.7)	0.58
1, *n* (%)	13 (26.5)	10 (45.5)	3 (11.1)	0.010
2, *n* (%)	12 (24.5)	6 (27.3)	6 (22.2)	0.75
3, *n* (%)	8 (16.3)	2 (9.1)	6 (22.2)	0.27
4, *n* (%)	9 (18.4)	2 (9.1)	7 (25.9)	0.16
5, *n* (%)	4 (8.2)	0 (0)	4 (14.8)	0.12

Values are expressed as mean ± standard deviation or *n* (%).

COPD: chronic obstructive pulmonary disease.

a Serum creatinine >1.5 mg/dl.

**Table 2:** Intraoperative data

Variable	Total	Ventral	Dorsal	*P*-value
	(*n *=* *49)	(*n *=* *22)	(*n *=* *27)	
Intraoperative data				
Ascending replacement, *n* (%)	11 (22.4)	7 (31.8)	4 (14.8)	0.19
Arch replacement, *n* (%)	38 (77.6)	15 (68.2)	23 (85.2)	0.19
Root surgery, *n* (%)	7 (14.3)	6 (27.3)	1 (3.7)	0.036
FET use, *n* (%)	33 (67.3)	13 (59.1)	20 (74.1)	0.36
FET diameter	27 (4)	27 (4)	27 (4)	0.62
FET length	120 (30)	120 (30)	120 (30)	0.68
Operative time, min	385.9 ± 94.7	400.2 ± 101.0	374.3 ± 89.5	0.35
CPB time, min	203.4 ± 52.4	209.2 ± 54.9	198.6 ± 50.9	0.49
Aortic cross-clamp time, min	106 (48)	101 (87)	107 (42)	0.83
Concomitant procedure				
AVR, *n* (%)	1 (2.0)	1 (4.6)	0 (0)	0.45
CABG, *n* (%)	1 (2.0)	1 (4.6)	0 (0)	0.47
TAP, *n* (%)	1 (2.0)	1 (4.6)	0 (0)	0.47

Values are expressed as mean ± standard deviation, median (interquartile range) or *n* (%).

AVR: aortic valve replacement; CABG: coronary artery bypass grafting; CPB: cardiopulmonary bypass; FET: frozen elephant trunk; TAP: tricuspid annuloplasty.

### Thoracoabdominal aortic morphology data

Thoracoabdominal aortic morphology data before and after surgery are shown in Table 3. The aortic diameter at the most dilated level was comparable between the 2 groups. The most dilated segment was at the same level of the most progressively expanded segment in 47 (97.5%) patients, and only 2 (4.1%) had the greatest difference in dilated aortic level compared with the most progressively expanded level. The spiral dissection pattern in the dorsal group was significantly higher than that in the ventral group (18.2% vs 66.7%, *P *=* *0.001).

**Table 3:** Postoperative thoraco-abdominal aortic morphology

Variable	Total	Ventral	Dorsal	*P*-value
	(*n *=* *49)	(*n *=* *22)	(*n *=* *27)	
Maximum dilated-level aortic morphology				
Preoperative diameter, mm	31.6 ± 3.6	31.6 ± 4.4	31.6 ± 2.9	0.99
Preoperative TL diameter, mm	13.8 ± 5.8	14.1 ± 7.0	13.6 ± 4.7	0.77
Preoperative FL diameter, mm	15.6 ± 5.4	14.5 ± 6.0	16.4 ± 4.8	0.22
Postoperative diameter, mm	33.8 ± 4.2	34.2 ± 4.8	33.5 ± 3.7	0.56
1-year postoperative diameter, mm	38.6 ± 5.8	40.3 ± 5.5	37.3 ± 5.9	0.070
True lumen morphology				
Concave type	24 (49.0)	12 (54.5)	12 (44.4)	0.56
Convex type	25 (51.0)	10 (45.5)	15 (55.6)	0.76
Helical pattern	22 (44.9)	4 (18.2)	18 (66.7)	0.001
Number of re-entry tears				
0, *n* (%)	1 (2.0)	0 (0)	1 (3.7)	1.000
1–3, *n* (%)	23 (46.9)	11 (50.0)	12 (44.4)	0.78
4–6, *n* (%)	23 (46.9)	10 (45.5)	13 (48.2)	0.77
>7, *n* (%)	2 (4.1)	1 (4.6)	1 (3.7)	1.000
Re-entry tear location				
Supra-aortic vessels	5 (10.2)	3 (13.6)	2 (7.4)	0.65
Descending aorta	15 (30.6)	8 (36.4)	7 (25.9)	0.54
Abdominal aorta	45 (91.8)	22 (100.0)	23 (85.2)	0.12
Iliac artery	40 (81.6)	18 (81.8)	22 (81.5)	1.000

Values are expressed as mean ± standard deviation or *n* (%).

FL: false lumen; TL: true lumen.

The number and location of re-entry tears between the groups were comparable.

**Figure 3:**
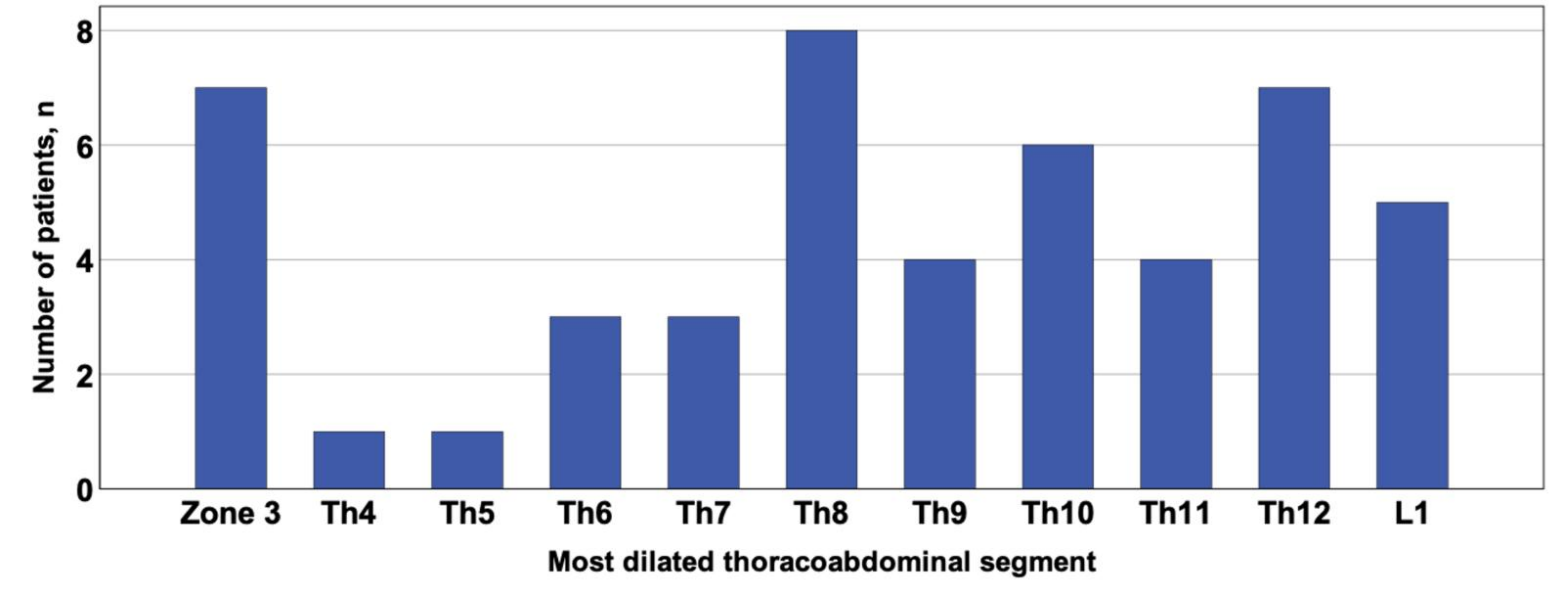
Distribution of the most dilated thoraco-abdominal aortic segment.

The distal aortic FL patency and >5 mm expansion rate are shown in Table 4. The patent FL rate was relatively higher in the ventral group than in the dorsal group, as derived from Fisher’s exact test (31.8% vs 11.1%; *P *=* *0.090). The incidence of expansion of the thoraco-abdominal aorta by 5 mm or more was significantly higher in the ventral group than in the dorsal group, as derived from Fisher’s exact test (90.9% vs 51.9%; *P *=* *0.009). The distribution of the most dilated thoraco-abdominal segment is shown in Fig. 3. Of the patients who had a ≥ 5 mm dilated segment at the 1-year postoperative CT, the incidences at the zone 3, Th8 and Th12 levels were relatively higher. The relationship between the most dilated or the largest thoraco-abdominal segment and size progression is shown in Fig. S1.

**Table 4:** Postoperative thoraco-abdominal aortic status

Variable	Total	Ventral	Dorsal	*P*-value
	(*n *=* *49)	(*n *=* *22)	(*n *=* *27)	
Patent FL, *n* (%)	10 (20.4)	7 (31.8)	3 (11.1)	0.090
Complete thrombosed FL, *n* (%)	1 (2.0)	0 (0)	1 (3.7)	1.000
Partial thrombosed FL, *n* (%)	37 (75.5)	15 (68.2)	22 (81.5)	0.33
≥5 mm dilation in 1-year, *n* (%)	34 (69.4)	20 (90.9)	14 (51.9)	0.009

Values are expressed as *n* (%).

FL: false lumen.

### Postoperative thoraco-abdominal aortic dilation risk factors

The univariable analysis revealed that the ventral type had a significantly higher OR for postoperative thoraco-abdominal aortic dilation [OR, 9.29; 95% confidence interval (CI), 1.81–47.77; *P *=* *0.008]. The multivariable logistic regression analysis demonstrated that the ventral type (OR, 6.01; 95% CI, 1.56–23.77; *P *=* *0.009), and the number of re-entry tears (OR, 1.91; 95% CI, 1.04–3.53; *P *=* *0.038) were independent prognostic factors for postoperative thoraco-abdominal aortic dilation (Table 5).

The data sets analysed in the current study are available from the corresponding author on reasonable request.

**Table 5:** Logistic regression analysis for postoperative thoraco-abdominal aortic dilation

	Univariable	*P*-value	Multivariable	*P*-value
Variable	OR (95% CI)		OR (95% CI)	
Age	0.99 (0.93–1.06)	0.77	–	–
Male	1.05 (0.29–3.81)	0.95	–	–
Hypertension	0.46 (0.12–1.74)	0.25	–	–
Body surface area	0.60 (0.04–8.98)	0.71	–	–
Aortic diameter	1.04 (0.85–1.27)	0.73	–	–
True lumen diameter	1.03 (0.93–1.15)	0.57	–	–
False lumen diameter	0.98 (0.87–1.09)	0.66	–	–
Helical pattern	0.61 (0.18–-2.08)	0.43	–	–
Concave type	1.50 (0.43–5.26)	0.53	–	–
Convex type	0.47 (0.12–1.87)	0.29	–	–
Number of re-entry tears	1.71 (0.98–2.98)	0.061	1.91 (1.04–3.53)	0.038
Re-entry tear at the descending aorta	2.18 (0.51–9.28)	0.29	–	–
Re-entry tear at the supra-aortic vessels	1.87 (0.19–18.27)	0.59	–	–
Number of visceral arteries arising from the FL	1.09 (0.71–1.69)	0.69	–	–
FET use	1.60 (0.45–5.69)	0.91	–	–
Arch replacement	1.65 (0.43–6.37)	0.47	–	–
Ascending aortic replacement	0.61 (0.16–2.34)	0.47	–	–
Root surgery	3.00 (0.33–27.40)	0.33	–	–
Ventral type	9.29 (1.81–47.77)	0.008	6.01 (1.56–23.77)	0.009

CI: confidential interval; FET: frozen elephant trunk; OR: odds ratio.

## DISCUSSION

Descending aortic remodelling is a key factor for favourable long-term outcomes after an operation for ATAAD. Several risk factors for poor late outcomes have been reported for acute TBAD; however, those in patients who undergo surgical repair for ATAAD have not been determined [17]. A patent FL is a known risk factor for poor late outcomes after ATAAD surgery; however, because aortic rupture or aortic reintervention is much lower than the rate of patent FL after ATAAD surgery, there are other risk factors for late-term aortic expansion and rupture [2, 5]. We found that abdominal aortic TL location after ATAAD repair may be a useful predictor of thoraco-abdominal aortic dilation.

Several risk factors, such as maximum aortic diameter or a large FL, have been reported in patients with acute TBAD [14]. Qin *et al.* reported that the number of visceral arteries arising from the FL is an independent risk factor of incomplete thrombosis after thoracic endovascular aortic repair (TEVAR) for acute TBAD [18]. However, because primary entry is usually excluded in patients who undergo ATAAD repair and the treatment range is more proximal in ATAAD repair than in TEVAR for TBAD, the mechanism of late-term aortic expansion may differ between patients who undergo ATAAD repair and those with acute TBAD or who undergo TEVAR.

Loewe *et al.* reported that the location of the primary entry tear negatively influenced the outcomes of acute TBAD [20]. In addition, the researchers showed that a primary entry tear at the inner curvature of the distal aortic arch is associated with a significant increase in the occurrence of complicated TBAD, although the precise mechanism of this phenomenon could not be explained. This result may be due to the same mechanism observed in our study. The primary entry tear at the inner curvature of the distal arch leads to the formation of the FL at the downside, which may increase the FL pressure because of the small outflow due to small re-entry tears originating from the minor branch.

Recently, 4-dimensional flow magnetic resonance imaging has been performed, particularly in patients with TBAD [10, 21]. Marlevi *et al.* reported that the FL ejection fraction, maximum systolic deceleration rate and the FL relative pressure derived from the 4D flow MRI analysis are independently correlated with aortic growth [10]. They showed that factors related to an increase in the FL pressure leads to aortic growth. The FL pressure is associated with the primary entry or re-entry tears, and primary entry is generally excluded after ATAAD repair; therefore, the re-entry tears play an important role in the increase in FL pressure after ATAAD repair. Stokes *et al.* suggested that intercostal branches arising from the FL influence FL pressure and wall shear stress, leading to FL dilation [22]. In addition, Sailer *et al.* suggested that an insufficient outflow or a mismatch between inflow and outflow may cause an increase in FL pressure, leading to either TL compression or FL expansion [16]. Based on these findings and indications, we hypothesized that the mismatch inflow and outflow are caused by the number and size of re-entry tears formed by the visceral branches and lumbar or intercostal arteries. For example, if the TL is in the ventral side and a large re-entry tear exists only at the dissection terminal (frequently at the iliac artery), the FL blood flow may not be able to return easily to the TL because of the small re-entry tears formed by the lumbar or intercostal arteries, and the FL pressure may increase. Therefore, we expected that the ventral type would correlate with the increase in FL pressure, because the re-entry tears of the ventral type, mostly from the lumbar or intercostal branches, were so small that the FL backward bloodstream may not have been able to easily return to the TL, resulting in increased FL pressure. Because the primary entry had a significantly larger influence on the FL pressure, the hypothesis is applicable only to patients with ATAAD who completed surgical repair. To simplify the classification, we set the evaluation point at the level of the renal artery, which is in the middle of the abdominal aorta, and defined the higher-risk group for aortic dilation as the ventral group.

Our main finding was that the abdominal TL location may predict thoraco-abdominal aortic dilation, which was associated with an increase in FL pressure. However, owing to the small study population, further analysis, such as a haemodynamic analysis using MRI, is required to elucidate the mechanism of thoraco-abdominal aortic dilation after surgical repair of ATAAD. Because this study population was small, we might have missed other independent risk factors for late-term thoraco-abdominal aortic dilation. Furthermore, we could not evaluate the sizes of the true and false lumens at the 1-year follow-up. Thus, late-term aortic size needs to be more precisely evaluated in further investigations.

Although the FET has been widely used for ATAAD repair, and several studies have reported favourable outcomes after the FET procedure, the rate of aortic reintervention after the FET procedure is unknown, and some patients experience thoraco-abdominal aortic dilation despite FET usage. Our study demonstrates the possibility of the mechanism for long-term aortic dilation after ATAAD repair with or without the FET.

### Limitations

This study has several limitations. First, the small sample size and the single-centre study could have caused selection bias. Also, the small sample size may have been inadequate to detect other independent risk factors for late-term thoraco-abdominal aortic dilation. Second, we excluded patients who died before undergoing the 1-year postoperative CT scan, which may have influenced the study outcomes. Third, because of the absence of contrast-enhanced CT scans at the 1-year postoperative follow-up, we could not evaluate the TL, FL and the precise aortic size during the follow-up period and possibly missed other risk factors for late-term thoraco-abdominal aortic dilation. Finally, although we hypothesized that aortic morphology is related to FL haemodynamic status and pressure, further investigation is needed to reveal the relationship between aortic morphology and haemodynamic status in the FL.

## CONCLUSION

The location of the TL in the abdominal aorta of patients with ATAAD may have a significant relationship with late-term thoraco-abdominal aortic dilation after ATAAD repair. The ventral type and the number of re-entry tears were independent predictors for late-term thoraco-abdominal aortic dilation in multivariable analysis.

## Supplementary Material

ivae063_Supplementary_Data

## Data Availability

The data sets analysed in the current study are available from the corresponding author on reasonable request.

## Notes

Presented at the 37^th^ Annual Meeting of the European Association for Cardio-Thoracic Surgery, Vienna, Austria, 4-7 October 2023.

## ABBREVIATIONS

ATAAD: Acute type A aortic dissection
CI: Confidential interval
CT: Computed tomography
FET: Frozen elephant trunk
FL: False lumen
OR: Odds ratio
TBAD: Type B aortic dissection
TL: True lumen
TEVAR: Thoracic endovascular aortic repair
