# Supplementary material for: Morphologic prognostic factor for thoracoabdominal aortic dilation after acute type A dissection repair
Source: Interdiscip Cardiovasc Thorac Surg. 2024 Apr 8;38(5):ivae063. doi: 10.1093/icvts/ivae063 (PMC11087929; doi:10.1093/icvts/ivae063)
Supplement: ivae063_Supplementary_Data [file ivae063_supplementary_data.pdf]

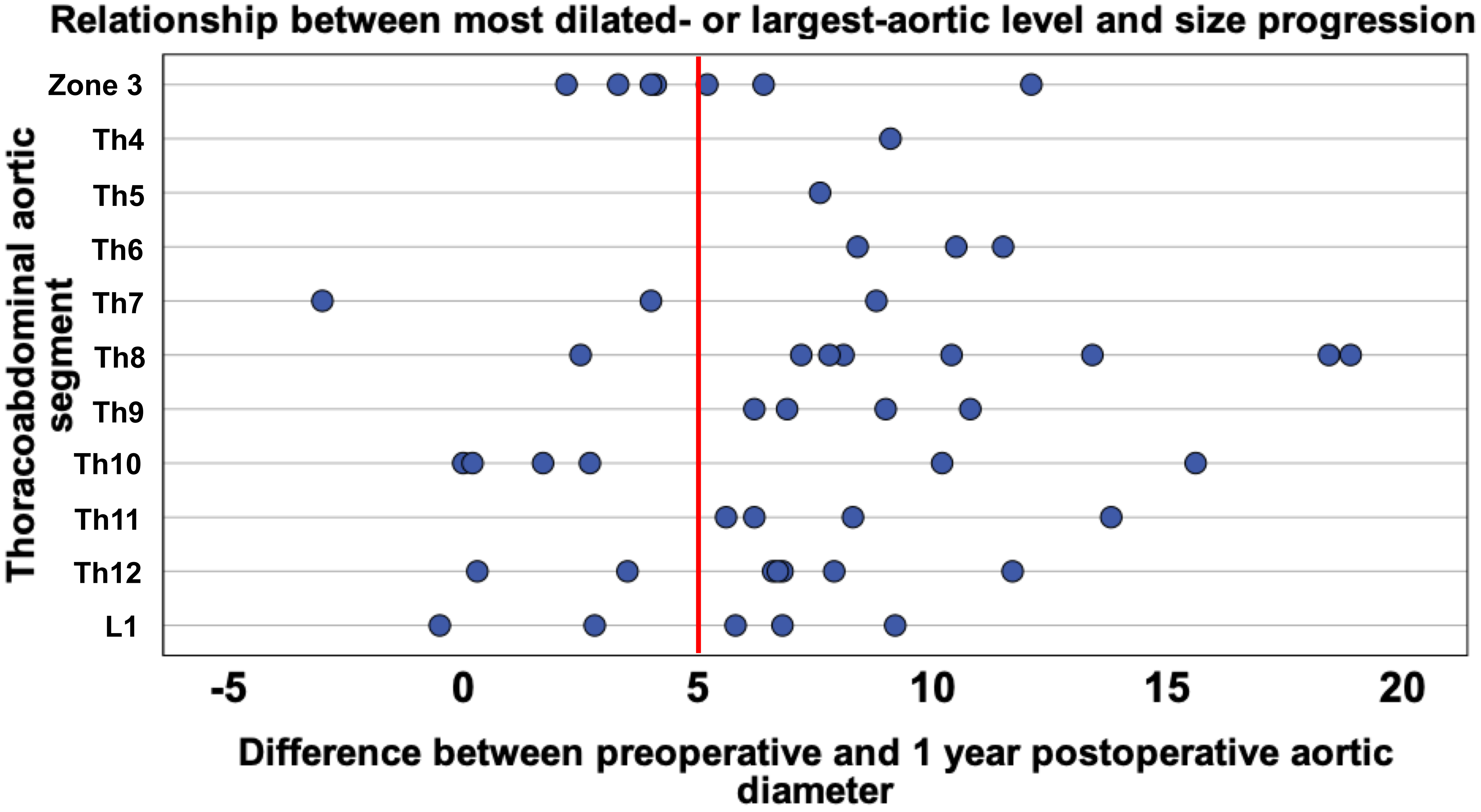


**Supplemental figure S1**: Relationship between the most dilated od largest-aortic level and aortic size progression.
